# The influence of left-behind experiences on depression in single-parent adolescents: the mediating role of family satisfaction and the moderating effects of exercise frequency

**DOI:** 10.3389/fped.2025.1573630

**Published:** 2025-05-14

**Authors:** Wang-Cheng Cen, Jing Liang, Xiao-Han Zhang, Wen-Jing Yan, Jun Qian

**Affiliations:** ^1^ School of Mental Health, Wenzhou Medical University, Wenzhou, China; ^2^ Department of Psychology, Hebei Normal University, Shijiazhuang, Hebei, China; ^3^ Renji College, Wenzhou Medical University, Wenzhou, China; ^4^ Zhejiang Provincial Clinical Research Center for Mental Health, Affiliated Kangning Hospital, Wenzhou Medical University, Wenzhou, China

**Keywords:** single-parent adolescents, depression, family satisfaction, exercise frequency, left-behind experiences, mediation

## Abstract

With the rise of rural-to-urban labor migration, the number of left-behind children in single-parent families has increased, raising concerns about their mental health. This study examines the impact of left-behind experiences on depression in single-parent left-behind adolescents, particularly focusing on the mediating role of family satisfaction and the moderating effects of exercise frequency and separation age. Using cross-sectional data from the China Psychological Health Guardian Project (CPHG), which includes 23,523 single-parent left-behind adolescents aged 12–18, we collected data on left-behind experiences, depressive symptoms, family satisfaction, exercise frequency, and separation age through questionnaires. Correlation analysis, multiple regression, and moderated mediation models were employed to test the hypotheses. The findings indicate that left-behind experiences significantly predict depressive symptoms (*t* = 7.77, *p* < 0.001), and family satisfaction serves as a significant mediator in this relationship (Indirect effect = 0.261, *t* = 4.971, *p* < 0.001). Additionally, exercise frequency moderates the effect of family satisfaction on depression (*B* = 0.42, *t* = 3.681, *p* < 0.001), where higher exercise frequency lessens the negative impact of low family satisfaction on depression. These results highlight the crucial roles of family satisfaction and exercise frequency in reducing depressive symptoms among single-parent left-behind adolescents, suggesting that emotional support and regular exercise can enhance their mental health.

## Highlights

• This study analyzed a large sample of 23,523 single-parent left-behind adolescents, providing robust findings.
• Higher exercise frequency moderates the effect of low family satisfaction on depression, reducing its negative impact.
• Left-behind experiences significantly increase depression levels in single-parent adolescents, with family satisfaction as a key mediator.

## 1 Introduction

In recent years, with the development of industrialization and urbanization, an increasing number of rural laborers have chosen to work away from home, leading to a large population of left-behind children. This phenomenon is particularly pronounced in single-parent families. According to attachment theory, the attachment bonds formed between children and their parents in early life have a profound impact on emotional regulation and mental health. The absence of parents can disrupt these attachment bonds, resulting in a lack of emotional support and increasing the emotional and psychological challenges faced by adolescents. Existing research has shown that single-parent left-behind adolescents, due to the absence of a complete family support system, are more likely to experience emotional distress and psychological pressure, particularly symptoms of depression (1). Depression not only affects an individual's emotional and social functioning but may also lead to self-harm or suicidal behaviors, making it crucial to explore its causes and prevention strategies (2).

Prolonged left-behind experiences, especially with extended periods of separation, may further weaken the role of emotional support from family members, exacerbating emotional distress and mental health problems in adolescents. Therefore, we hypothesize that the duration of left-behind experiences will significantly affect the depression levels of single-parent adolescents (H1), specifically, the longer the duration of being left behind, the more severe the depressive symptoms. This hypothesis aims to deepen the understanding of the psychological effects of left-behind experiences on single-parent adolescents and to provide a theoretical basis for psychological intervention and support for this group.

### 1.1 The mediating role of family satisfaction

The family is an essential source of psychological support for adolescents, and family satisfaction plays a particularly important role in the psychological development of single-parent adolescents. According to family systems theory, the family is viewed as an emotional unit, with interactions between members having mutual effects. Adolescents' mental health is not only influenced by individual factors but is also deeply affected by the overall atmosphere of family relationships. Family satisfaction refers to an individual's overall evaluation of family relationships, including emotional support, communication, and trust. Studies have shown that family satisfaction is closely related to adolescents' mental health. In situations where parents are long-term absentees, decreased family satisfaction significantly increases the risk of depression (3, 4). For single-parent left-behind adolescents, the absence of parents may lead to a reduction in emotional support, thus lowering family satisfaction and increasing the occurrence of depressive symptoms (5).

Hence, we propose Hypothesis H2: Family satisfaction mediates the relationship between left-behind experiences and depression in single-parent adolescents, whereby left-behind experiences increase depressive symptoms by lowering family satisfaction.

### 1.2 The moderating role of separation age

The psychological impact of left-behind experiences varies significantly across different age groups, closely related to the emotional needs and coping abilities at specific developmental stages. According to psychosocial development theory, a key task during adolescence is the establishment of identity and the development of intimate relationships, both of which require emotional support from parents. Adolescents have a strong emotional dependence on their parents during this period, and thus, the absence of parents during adolescence may have a more profound impact on mental health (6, 7). Compared to earlier separations, left-behind experiences occurring at later developmental stages may have more severe negative effects on family relationships and psychological well-being, such as lowering family satisfaction and exacerbating depressive symptoms (8).

Based on this research, we hypothesize that separation age moderates the relationship between the duration of left-behind experiences and both family satisfaction and depression levels. Therefore, we propose Hypothesis H3: Separation age moderates the relationship between the duration of left-behind experiences and family satisfaction, as well as the relationship between the duration of left-behind experiences and depressive symptoms. Specifically, later separation ages will strengthen the negative impact of left-behind experiences on family satisfaction and worsen the effect on depressive symptoms.

### 1.3 The moderating role of exercise frequency

Exercise frequency is an important behavioral factor influencing adolescents' mental health. Research shows that regular physical exercise can effectively reduce the occurrence of depressive symptoms by enhancing self-esteem and emotional regulation abilities (9, 10).

The literature indicates that exercise frequency plays a moderating role between family satisfaction and depression, as well as between left-behind experiences and depression. Higher exercise frequency can enhance the protective effect of family satisfaction against depressive symptoms, meaning that even when family satisfaction is low, adolescents can maintain better mental health through exercise (11). Furthermore, exercise frequency may mitigate the risk of depression caused by prolonged parental absence. The emotional improvement and social interaction brought about by exercise help adolescents better cope with the negative emotions associated with left-behind experiences (12).

Based on the above discussion, we propose Hypothesis H4: Exercise frequency moderates the relationship between family satisfaction and depression, as well as the relationship between the duration of left-behind experiences and depressive symptoms. Specifically, higher exercise frequency can weaken the negative effect of low family satisfaction on depression and reduce the negative impact of left-behind experiences on depressive symptoms.

Previous research has examined the mental health of left-behind children, but most studies have either focused on general left-behind children or two-parent families. While these studies have provided valuable insights into the psychological effects of parental absence, particularly with respect to depressive symptoms, they have largely overlooked the unique experiences of single-parent left-behind adolescents. Although some studies have addressed the role of family satisfaction and exercise in mental health, there is still a lack of research specifically focusing on single-parent left-behind adolescents, a vulnerable group that may face distinct emotional challenges due to the absence of one parent in the family structure.

In summary, our study constructs a mediated moderation model to explore how family satisfaction, separation age, and exercise frequency affect the relationship between left-behind experiences and depression in single-parent adolescents. This model offers a more nuanced understanding of the psychological impacts of left-behind experiences by specifically focusing on single-parent left-behind adolescents, a group that has been under-researched in existing studies. By examining the mediating role of family satisfaction and the moderating effects of exercise frequency, this study aims to provide theoretical support and intervention ideas that can improve the mental health of this vulnerable population.

## 2 Method

### 2.1 Research design and sample

This study is based on data from the China Psychological Health Guardian Project (CPHG) and employs a cross-sectional research design. The goal is to explore the impact of the duration of left-behind experiences on depression in single-parent adolescents aged 12–18 years, while examining the mediating role of family satisfaction and the moderating effects of exercise frequency and separation age. Data collection for this project took place from October 2022 to May 2023 and covered multiple centers in Nanchong City, Sichuan Province, focusing on underrepresented children in social and psychological care systems, including orphans, de facto unsupervised children, single-parent children, and left-behind children. The total sample size was 249,772 children.

For analysis, we selected the left-behind children group, ultimately screening out 23,523 eligible single-parent left-behind adolescents. Left-behind children were defined as those separated from one or both parents for six months or more due to the parents working away from home. The participants' ages ranged from 12 to 18 years, and all data were collected through questionnaires, covering demographic variables such as gender, age, family economic status, and variables related to the duration and age of the left-behind experience.

### 2.2 Measurement methods

#### 2.2.1 Duration of left-behind experiences

The duration of left-behind experiences was measured based on the length of time participants were separated from their parents. In the questionnaire, participants were asked to report the time period of separation due to parental work, categorized into the following intervals: 6 months to 1 year, 1–2 years, 2–4 years, 5–10 years, and more than 10 years. This variable was used to quantify the intensity of left-behind experiences and to assess its impact on adolescents' depression levels.

#### 2.2.2 Depression levels

Depressive symptoms were assessed using the widely employed Center for Epidemiological Studies Depression Scale (CES-D). The scale consists of 20 items that measure the frequency of depressive symptoms experienced by participants over the past week. Each item is rated on a four-point Likert scale ranging from “rarely” (0 points) to “almost every day” (3 points). The total score is obtained by summing all items, with higher scores indicating more severe depressive symptoms. The CES-D scale has demonstrated good reliability and validity in adolescent populations and is suitable for screening depressive symptoms in non-clinical groups. In this study, the Cronbach's alpha coefficient for the CES-D scale was 0.949.

#### 2.2.3 Family satisfaction

Family satisfaction was assessed using a single question asking participants to self-evaluate their satisfaction with family relationships. Responses were rated on a five-point Likert scale ranging from “very satisfied” to “very dissatisfied.” This variable was used to analyze the mediating role of family satisfaction in the relationship between left-behind experiences and depressive symptoms, i.e., whether family satisfaction serves as a bridge between the two.

#### 2.2.4 Exercise frequency

Exercise frequency was measured with a single question assessing how often participants engage in physical activity each week, with response options including “never,” “weekends only,” “occasionally (1–2 times per week),” “frequently (3–4 times per week),” and “every day.” Exercise frequency was treated as a moderating variable to examine its moderating effect on the relationship between left-behind experiences and depressive symptoms, aiming to explore whether physical exercise can alleviate the negative psychological impacts of left-behind experiences.

#### 2.2.5 Separation age

Separation age was measured by assessing the age at which participants first experienced separation from their parents. The age groups were divided into infancy (0–1.5 years), toddlerhood (1.5–3 years), preschool age (3–6 years), school age (6–12 years), and adolescence (12–18 years). This variable was introduced to examine the differential effects of left-behind experiences at different developmental stages on depressive symptoms, i.e., whether separation at different ages moderates the impact of left-behind experiences on depression levels.

#### 2.2.6 Control variables

Age, gender, and family economic status were included as control variables to reduce the interference of confounding variables on the results. Age was a continuous variable, recording the participants' actual age. Gender was a categorical variable, with male coded as 1 and female coded as 0. Family economic status was measured based on participants' subjective evaluation of their family's economic condition, ranging from “poor” to “wealthy,” to control for the potential impact of economic background on depressive symptoms.

### 2.3 Data processing

Statistical analysis, including descriptive statistics, reliability analysis, correlation analysis, and binary logistic regression, was conducted using SPSS 26.0. Hayes' PROCESS macro for SPSS was employed to perform moderated mediation effect analysis.

## 3 Results

### 3.1 Descriptive statistics

Descriptive statistical analysis was conducted on the key variables within the sample of single-parent left-behind adolescents. The total sample consisted of 23,523 participants, with a relatively balanced gender distribution: 49.2% male and 50.8% female. The participants' ages ranged from 12 to 18 years, with a mean age of 14.31 years (*SD* = 1.71). Additionally, the mean subjective evaluation of family economic status was 1.96 (*SD* = 0.589), indicating that most participants considered their family's economic situation to be of average level.

### 3.2 Correlation analysis

The correlation analysis revealed a significant positive correlation between the duration of left-behind experiences and depression scores (*r* = 0.127, *p* < 0.01), while the separation age was significantly negatively correlated with depression scores (*r* = −0.067, *p* < 0.01). Family satisfaction was significantly negatively correlated with depression scores (*r* = −0.391, *p* < 0.01), and exercise frequency was also significantly negatively correlated with depression scores (*r* = −0.125, *p* < 0.01). Furthermore, gender (*r* = −0.175, *p* < 0.01) and age (*r* = 0.155, *p* < 0.01) were significantly correlated with depression scores, whereas family economic status was not significantly correlated with depression scores (*r* = −0.005, *p* > 0.05). Specific correlation coefficients are presented in Table 1.

**Table 1:** Descriptive statistics and correlations among the variables.

Variables	*M*	*SD*	CES-D scores	Separation age	Duration of left-behind	Family satisfaction	Exercise frequency	Gender	Age	Economic status
CES-D scores	11.35	11.826	1							
Separation sge	3.24	1.304	−0.067**	1						
Duration of left-behind	2.75	1.423	0.127**	−0.246**	1					
Family satisfaction	3.63	1.027	−0.391**	0.074**	−0.125**	1				
Exercise frequency	3.11	1.128	−0.125**	0.027**	−0.025**	0.075**	1			
Gender	—	—	−0.175**	0.041**	0.009	0.097**	0.133**	1		
Age	14.4	1.722	0.155**	0.042**	0.089**	−0.078**	−0.057**	0.016**	1	
Economic status	1.93	0.58	−0.005	0.049**	−0.083**	0.086**	0.025*	−0.041**	−0.009	1

**p < 0.01. * *p* < 0.05.

### 3.3 Mediation effect test

Descriptive statistics indicated correlations between depression levels, family satisfaction, gender, age, and economic status. Therefore, gender, age, and economic status were included as control variables. After standardizing the data and controlling for gender, age, and economic status, we used Model 4 of the PROCESS macro to test the mediating effect of family satisfaction.

**Figure 1:**
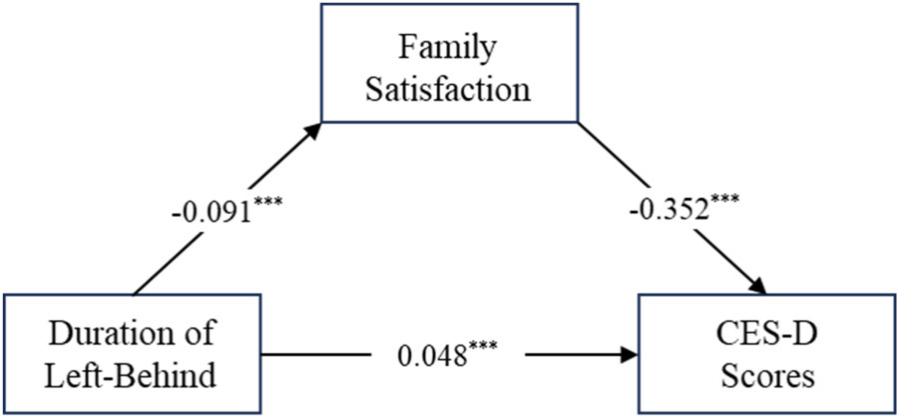
The mediating role of family satisfaction.

As shown in Tables 2, 3, and Figure 1, the positive predictive effect of the duration of left-behind experiences on depression levels was significant (*p* < 0.001). After introducing the mediating variable of family satisfaction, the direct predictive effect of the duration of left-behind experiences on depression levels remained significant (*p* < 0.001). The total effect of the duration of left-behind experiences on depression levels was 0.653 (*t* = 7.77, *p* < 0.001), and the direct effect was 0.392 (*t* = 4.971, *p* < 0.001), indicating that family satisfaction partially mediated the relationship between the duration of left-behind experiences and depression levels. Specifically, the duration of left-behind experiences significantly negatively predicted family satisfaction (*β* = −0.091, *t* = −8.72, *p* < 0.001), and family satisfaction significantly negatively predicted depression levels (*β* = −0.352, *t* = −36.18, *p* < 0.001). The Bootstrap test revealed an estimated indirect effect of 0.261 (Boot SE = 0.032), with a 95% confidence interval excluding 0, indicating that the indirect effect was significant.

**Table 2:** Regression models analysis.

Predictor variables	Model1		Model2		Model3	
	(Depression levels)		(Family satisfaction)		(Depression levels)	
	*β*	*t*	*β*	*t*	*β*	*t*
Gender	−0.176	−17.093***	0.097	9.36***	−0.141	−14.654***
Age	0.110	10.707***	−0.063	−6.037***	0.088	9.139***
Economic status	0.01	0.956	0.074	7.11***	0.036	3.721***
Duration of left-behind	0.08	7.77***	−0.091	−8.72***	0.048	4.971***
Family satisfaction					−0.352	−36.18***
*R* ^2^	0.051	0.029	0.171			
*F*	121.2***	66.16***	372.83***			

***p < 0.001.

**Table 3:** Total, direct, and indirect effects analysis.

Effect type	Effect	SE	*t*	*p*	LLCI	ULCI
Total effect	0.653	0.084	7.77	<0.001	0.488	0.818
Direct effect	0.392	0.079	4.971	<0.001	0.237	0.547
Indirect effect	0.261	0.032	/	/	0.199	0.323

### 3.4 Moderated mediation effect test

**Figure 2:**
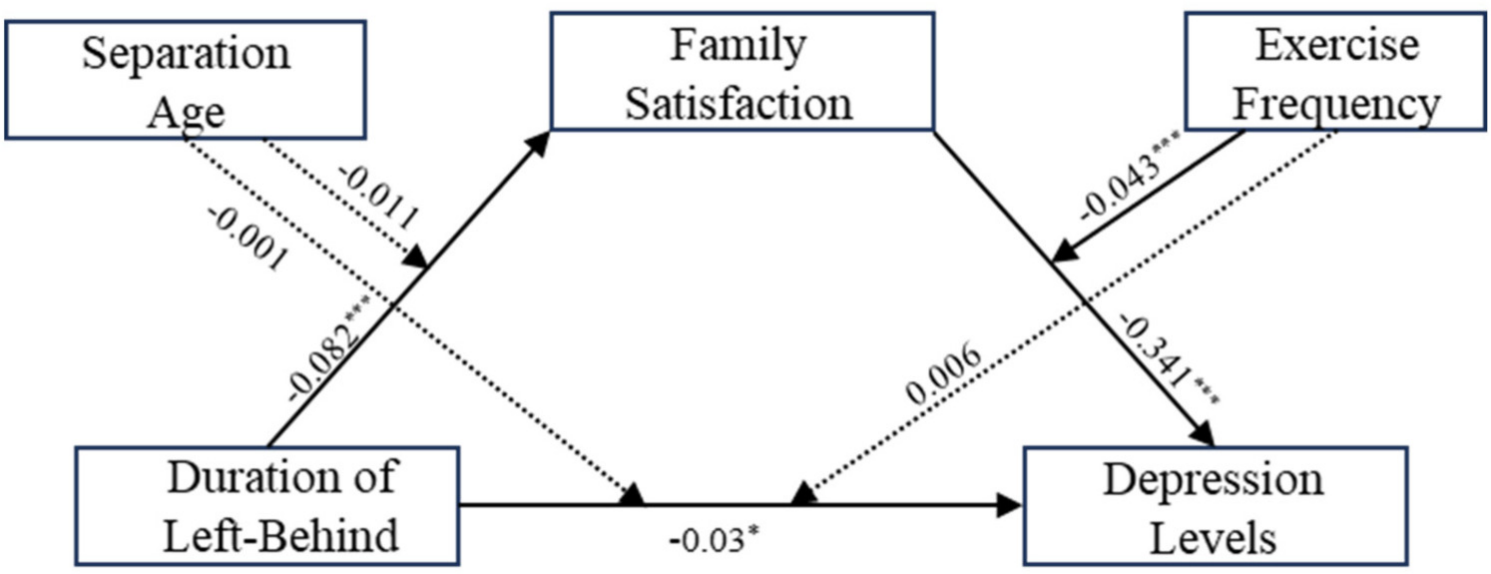
Moderated mediation model. The solid lines represent significant direct relationships between variables, while the dashed lines represent non-significant relationships. The solid lines show that the duration of left-behind experiences significantly affects family satisfaction and depression levels, that family satisfaction significantly influences depression levels, and that exercise frequency moderates the relationship between family satisfaction and depression levels.

Building on the mediation effect analysis, we used Model 29 of the PROCESS macro to examine the moderating effects of exercise frequency and separation age, controlling for gender, age, and economic status. As shown in Table 4 and Figure 2, the interaction between family satisfaction and exercise frequency significantly predicted depression levels (*β* = 0.043, *t* = 3.787, *p* < 0.001). However, other paths, such as the interaction between the duration of left-behind experiences and exercise frequency, were not significant (*p* > 0.05), indicating that exercise frequency significantly moderated the effect of family satisfaction on depression levels.

**Table 4:** The moderating roles of separation age and exercise frequency.

Predictor variables	Model1		Model2	
	(Family Satisfaction)		(Depression Levels)	
	*β*	*t*	*β*	*t*
Gender	0.097	7.872***	−0.125	−11.001***
Age	−0.073	−5.667***	0.092	7.733***
Economic status	0.074	6.072***	0.037	3.29**
Duration of left-behind (*X*)	−0.082	−6.33***	0.03	2.511*
Separation age (*W*)	0.046	3.582**	−0.024	−1.987
Left-behind duration × Separation age (*X* × *W*)	−0.011	−0.893	−0.001	−0.082
Family satisfaction (*M*)			−0.341	−29.229***
Exercise frequency (*Z*)			−0.072	−6.361***
Left-behind duration × Exercise frequency (*X* × *Z*)			0.006	0.503
Family satisfaction × Exercise frequency (*M* × *Z*)			0.043	3.787***
*R* ^2^	0.033	0.177		
*F*	35.393***	132.179***		

***p < 0.001. **p < 0.01. *p < 0.05.

**Figure 3:**
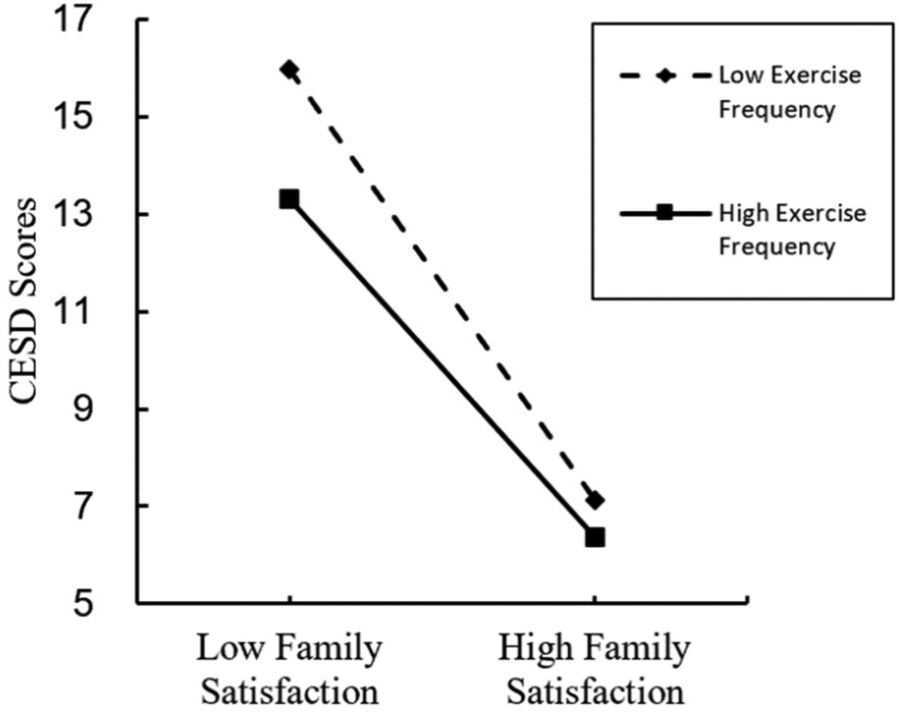
The moderating effect of exercise frequency on the relationship between family satisfaction and depression. The dashed line represents adolescents with low exercise frequency, while the solid line represents those with high exercise frequency. At lower levels of family satisfaction, adolescents with low exercise frequency exhibit considerably higher depression scores compared to their high exercise frequency counterparts. As family satisfaction increases, depression levels decline in both groups, with a more pronounced reduction observed among adolescents with high exercise frequency.

A simple slope analysis was conducted to examine the moderating effect of high/low exercise frequency. As illustrated in Figure 3, under low exercise frequency, the effect of family satisfaction on depression levels was significant (simple slope_low_ = −4.392, *t* = −23.923, *p* < 0.001), while under high exercise frequency, the negative effect of family satisfaction on depression levels was still significant but weakened (simple slope_high_ = −3.447, *t* = −18.514, *p* < 0.001). This indicates that exercise frequency significantly moderated the effect of family satisfaction on depression levels, with higher exercise frequency reducing the strength of this negative effect.

## 4 Discussion

This study systematically analyzed the impact of left-behind experiences on depression levels in single-parent adolescents, yielding the following key conclusions: First, the longer the duration of left-behind experiences, the higher the level of depression, confirming Hypothesis H1. Second, family satisfaction mediated the relationship between left-behind experiences and depression, where lower family satisfaction further exacerbated depressive symptoms, supporting Hypothesis H2. Third, although Hypothesis H3 proposed that the separation age might moderate this relationship, no significant moderating effect was found. Finally, exercise frequency significantly moderated the impact of family satisfaction on depression levels, partially validating Hypothesis H4, as higher exercise frequency mitigated the depressive effects of low family satisfaction.

### 4.1 Left-behind duration, family satisfaction, and depression levels

This study found a significant positive correlation between the duration of left-behind experiences and depression levels in single-parent adolescents, confirming Hypothesis H1. That is, the longer the left-behind period, the more severe the depressive symptoms. This finding aligns with existing literature, suggesting that prolonged separation from parents weakens the emotional support available to adolescents, thereby intensifying emotional distress (13, 14). The lack of emotional support not only makes adolescents feel isolated and helpless but also affects their self-esteem and emotional regulation abilities, particularly when they lack the presence of both parents for adequate guidance and support (15). However, merely exploring the direct relationship between the duration of left-behind experiences and depression does not fully reveal the complexity of the issue. The results further indicate that family satisfaction plays a crucial mediating role, suggesting that left-behind experiences indirectly exacerbate depressive symptoms by weakening the stability of family relationships and emotional support, thus validating Hypothesis H2.

Specifically, as the duration of left-behind experiences increases, communication and trust between adolescents and their parents gradually weaken, leading to dissatisfaction with family relationships, which in turn increases the risk of depression (16, 17). As the core support system for emotional development, the family plays a critical role in providing security and belonging. When emotional support within the family is insufficient, adolescents may struggle to regulate their emotions, further deteriorating their social relationships and mental health. Therefore, the impact of left-behind experiences on depression is not isolated but occurs indirectly by reducing family satisfaction and emotional support within the family. This mediating mechanism highlights the central role of family relationships and underscores the importance of enhancing emotional support within the family to alleviate depressive symptoms.

### 4.2 The moderating role of separation age

This study hypothesized that separation age would significantly moderate the relationship between the duration of left-behind experiences, family satisfaction, and depression levels. However, the results showed that the moderating effect was not significant, differing from previous findings that suggested adolescence is a particularly sensitive period for the psychological impact of left-behind experiences (6, 7).

We acknowledge that this null result may be due to several potential methodological factors. First, the broad age categories used to measure separation age (e.g., Infancy, Early Childhood, Adolescence) may not have captured the developmental nuances across different stages. Specifically, grouping a wide age range together may have diluted the potential impact of specific age periods. Research suggests that different developmental stages may have varying levels of vulnerability to parental separation, and the broad categorization of separation age might not have accurately reflected these differences (18). Therefore, future studies could refine the measurement by using more precise age intervals, which would allow for a better understanding of how separation age at specific developmental stages impacts adolescent mental health.

Second, although the sample size in this study is large, the complexity of the model—particularly the inclusion of multiple potential moderating and mediating variables—may have affected the ability to detect the interaction effect between separation age and other variables, such as family satisfaction. Research suggests that complex models with many interacting variables might require even more statistical power to detect subtle effects (19). Therefore, insufficient power to detect interaction effects could have contributed to the lack of a significant result in our study.

Moreover, while the effect of separation age was hypothesized to moderate the relationship between family satisfaction and depression, it is possible that other factors, such as peer relationships or school environments, might have played a more dominant role in influencing depression symptoms. Previous research has suggested that social support networks and coping strategies can significantly impact the mental health of adolescents, sometimes overshadowing the effect of family dynamics (20, 21). These factors may have weakened the potential moderating effect of separation age in this context.

### 4.3 The moderating role of exercise frequency

The results of this study demonstrated that exercise frequency significantly moderated the relationship between family satisfaction and depression levels, supporting part of Hypothesis H4. Higher exercise frequency was found to weaken the negative impact of low family satisfaction on depressive symptoms. As illustrated by the simple slope analysis in Figure 3, under conditions of low family satisfaction, adolescents with lower exercise frequency exhibited significantly higher depression scores compared to those with higher exercise frequency. This indicates that higher exercise frequency effectively alleviated depressive symptoms caused by low family satisfaction. This finding is consistent with research by (10, 11), which suggested that physical exercise helps improve self-esteem and emotional state, especially when family support is insufficient, making exercise a vital tool for adolescents to cope with negative emotions.

However, for the other part of Hypothesis H4, which proposed that exercise frequency would moderate the relationship between the duration of left-behind experiences and depression levels, no significant moderating effect was found in this study. This contrasts with the findings of (12), who argued that exercise could serve as a protective factor against the negative psychological effects of long-term left-behind experiences.

This discrepancy may stem from the complex and multifaceted nature of the negative psychological impact of left-behind experiences, which involve not only emotional deprivation but also attachment disruption and long-term familial separation. These effects often lead to a profound sense of emotional loneliness and social isolation, which exercise may not be able to fully mitigate. While exercise can help regulate short-term emotional responses and provide immediate stress relief (22), the deep emotional wounds caused by long-term parental absence are more difficult to address through physical activity alone. Research indicates that left-behind children often experience significant disruptions in their attachment systems, which may create long-term feelings of insecurity and a diminished sense of belonging, which are less likely to be alleviated by physical exercise (23).

Moreover, left-behind experiences tend to have lasting effects on identity formation and emotional regulation, which are critical during adolescence. The prolonged lack of emotional support from parents can lead to entrenched emotional patterns that require more complex interventions, such as therapeutic support, rather than being solely buffered by physical activity (24). Exercise may contribute positively to emotional regulation, but it cannot replace the deep emotional bonds and consistent support that children require from their primary caregivers. Therefore, while exercise appears to be a helpful tool in managing the emotional consequences of low family satisfaction, it may not provide enough protection against the deeper, more ingrained psychological effects of being left behind.

### 4.4 Study limitations

This study has several limitations. First, although the moderating role of exercise frequency in the relationship between family satisfaction and depression symptoms was examined, the moderating effect on the relationship between left-behind experiences and depression was not significant. This may be related to the specificity of the sample, the type, intensity, and duration of exercise. Future research could refine the exercise categories and explore how different types of physical activity affect adolescent mental health.

Second, this study primarily focused on family satisfaction and exercise frequency as factors influencing left-behind experiences. However, other potential moderators or mediators, such as social support networks, school environments, and peer relationships, also play a critical role in adolescent mental health. Future research should consider a broader range of environmental and social factors to comprehensively understand the complex mechanisms through which left-behind experiences impact adolescent mental health.

Additionally, the use of a single-item measure for family satisfaction may have limited the reliability and validity of this construct. Future studies should consider using multi-item scales to more accurately assess family satisfaction and its impact on adolescent mental health.

## 5 Conclusion

This study systematically analyzed the impact of left-behind experiences on depression in single-parent adolescents through a mediation and moderation model, highlighting the mediating role of family satisfaction. The results showed that the longer the duration of being left behind, the lower the family satisfaction, which in turn aggravated depressive symptoms. Family satisfaction played a crucial role in this process, confirming its importance in alleviating the negative psychological impacts.

Additionally, exercise frequency significantly moderated the relationship between family satisfaction and depression, with higher exercise frequency helping to reduce depressive symptoms caused by low family satisfaction. Although the moderating effect of separation age was not significant, the study provides practical recommendations for improving the mental health of single-parent left-behind adolescents, emphasizing the importance of emotional support and regular physical exercise.

## Data Availability

The original contributions presented in the study are included in the article/Supplementary Material, further inquiries can be directed to the corresponding author.
